# Larval Zebrafish Lateral Line as a Model for Acoustic Trauma

**DOI:** 10.1523/ENEURO.0206-18.2018

**Published:** 2018-08-30

**Authors:** Phillip M. Uribe, Beija K. Villapando, Kristy J. Lawton, Zecong Fang, Dmitry Gritsenko, Ashwin Bhandiwad, Joseph A. Sisneros, Jie Xu, Allison B. Coffin

**Affiliations:** 1 Department of Integrative Physiology and Neuroscience, Washington State University, Vancouver, WA 98686; 2 College of Arts and Sciences, Washington State University, Vancouver, WA 98686; 3 Mechanical Engineering Department, Washington State University, Vancouver, WA 98686; 4 Department of Mechanical and Industrial Engineering, University of Illinois at Chicago, Chicago, IL 60607; 5 Department of Psychology, University of Washington, Seattle, WA 98195

**Keywords:** acoustic trauma, hair cell, hearing loss, lateral line, zebrafish

## Abstract

Excessive noise exposure damages sensory hair cells, leading to permanent hearing loss. Zebrafish are a highly tractable model that have advanced our understanding of drug-induced hair cell death, yet no comparable model exists for noise exposure research. We demonstrate the utility of zebrafish as model to increase understanding of hair cell damage from acoustic trauma and develop protective therapies. We created an acoustic trauma system using underwater cavitation to stimulate lateral line hair cells. We found that acoustic stimulation resulted in exposure time- and intensity-dependent lateral line and saccular hair cell damage that is maximal at 48–72 h post-trauma. The number of TUNEL+ lateral line hair cells increased 72 h post-exposure, whereas no increase was observed in TUNEL+ supporting cells, demonstrating that acoustic stimulation causes hair cell-specific damage. Lateral line hair cells damaged by acoustic stimulation regenerate within 3 d, consistent with prior regeneration studies utilizing ototoxic drugs. Acoustic stimulation-induced hair cell damage is attenuated by pharmacological inhibition of protein synthesis or caspase activation, suggesting a requirement for translation and activation of apoptotic signaling cascades. Surviving hair cells exposed to acoustic stimulation showed signs of synaptopathy, consistent with mammalian studies. Finally, we demonstrate the feasibility of this platform to identify compounds that prevent acoustic trauma by screening a small redox library for protective compounds. Our data suggest that acoustic stimulation results in lateral line hair cell damage consistent with acoustic trauma research in mammals, providing a highly tractable model for high-throughput genetic and drug discovery studies.

## Visual Abstract

**Figure:**
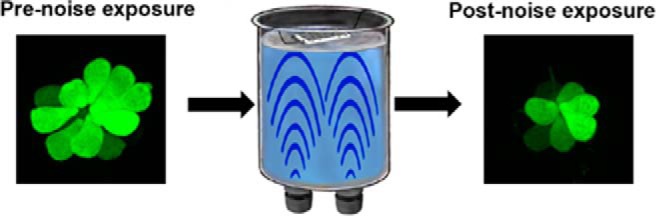

## Significance Statement

Noise overexposure damages hair cells, leading to permanent hearing loss. A critical step in understanding and preventing noise-induced hearing loss (NIHL) is establishing an accessible *in vivo* model to test for genetic and chemical modulators of noise damage. We developed a novel acoustic trauma system, using cavitation, to stimulate and damage zebrafish lateral line hair cells. We demonstrate that acoustic stimulation damages zebrafish lateral line hair cells in an exposure time- and intensity-dependent manner, consistent with acoustic trauma research in mammals. This novel system provides a model for *in vivo*, real-time studies of noise exposure, and for rapid discovery of chemical and genetic modulators of acoustic trauma-induced hair cell damage.

## Introduction

Noise-induced hearing loss (NIHL) is the most common cause of hearing loss and the second most common occupational illness in the United States (Bureau of Labor Statistics, 2006). The effects of NIHL are comorbid with depression, social isolation, and functional decline (Mick et al., 2014). Noise exposure can lead to permanent hearing impairment as the result of damage to mechanosensory hair cells within the cochlea. Despite identification of cell death cascades associated with acoustic trauma, there is a lack of information about damage onset and progression. Further, although many candidate protective targets have been identified in rodents, no FDA-approved therapy exists and target innovation has been slow (Le Prell et al., 2007). Thorough characterization of the progression of hair cell degeneration following noise exposure would represent a major advancement toward understanding and preventing NIHL. This goal necessitates the development of a more accessible platform where viable hair cells can be rapidly assessed *in vivo*.

Overexposure to intense noise causes hair cell damage 1–14 d after exposure and results in permanent threshold shifts (Wang et al., 2002). A primary biochemical mechanism of noise-induced hair cell damage is overproduction of reactive oxygen species (ROS; Henderson et al., 2006). Immediately following noise trauma, hair cells exhibit increased ROS production that persists up to 10 d. Antioxidant therapies reduce NIHL in rodent models, which is consistent with a ROS-mediated hair cell death mechanism (Yamane et al., 1995; Ohlemiller, 2008). ROS overproduction can lead to apoptosis or regulated necrosis, and both processes have been observed in noise-exposed hair cells (Hu et al., 2002; Nicotera et al., 2003).

In contrast, acoustic trauma that only induces temporary threshold shifts does not result in direct hair cell loss. Exposure to moderately intense noise causes cochlear synaptopathy, often leading to accelerated age-related hearing loss (Kujawa and Liberman, 2009; Sergeyenko et al., 2013; Fernandez et al., 2015). In humans, synaptopathy likely contributes to tinnitus, hyperacusis, and difficulty processing speech in noise (Liberman and Kujawa, 2017). Neurotrophic therapies such as neurotrophin-3 and BDNF show promise in regenerating cochlear synapses after noise damage (Sly et al., 2016; Suzuki et al., 2016), but more studies are needed to understand mechanisms of synaptopathy and develop preventative therapies.

The zebrafish lateral line, which contains hair cells homologous to those of the mammalian inner ear, represents a tractable model for studies of hair cell function, damage, and protection (Harris et al., 2003; Owens et al., 2008; Nicolson, 2017). The lateral line is located on the body surface and encodes mechanosensory stimuli, providing the sense of “distant touch” (Dijkgraaf, 1963). Our understanding of chemical ototoxicity has greatly benefited from the utility of larval zebrafish as a model (Coffin et al., 2013; Esterberg et al., 2013, 2014). Larval zebrafish are an ideal model for hair cell damage studies due to large clutch sizes, optical transparency, and small body size, which allow for treatments to be performed in well plates (Mathias et al., 2012). These characteristics enable *in vivo* imaging of damage processes and allow for rigorous quantitative exploration of the noise damage parameter space using a large number of animals. Additionally, large scale drug screens are possible using the larval zebrafish lateral line. These studies have identified novel otoprotective drug candidates that can preserve hearing in rodent models of ototoxicity, demonstrating that compounds initially identified in zebrafish translate to mammalian systems (Owens et al., 2008; Uribe et al., 2015; Chowdhury et al., 2018).

Here, we introduce a novel process to study acoustic trauma in larval zebrafish. Other groups have used underwater speakers to damage inner ear hair cells of fish but there have been no reports of an acoustic stimulus to damage lateral line hair cells (Popper and Fay 1973; Schuck and Smith, 2009). Our process uses cavitation, which occurs when dissolved gases in a fluid interact with ultrasonic waves resulting in oscillation of microbubbles. Microbubbles reach a maximum size and implode, emitting broadband shockwaves (Leighton, 1994). We demonstrate that underwater acoustic stimulation likely produced by cavitation specifically damages lateral line hair cells in a time- and intensity-dependent manner and is prevented by antioxidant therapy, consistent with mammalian models of acoustic trauma. Zebrafish represent a novel platform for understanding the timing of events in noise-damaged hair cells and for future high-throughput drug discovery studies aimed at preventing noise-induced hair cell damage.

## Materials and Methods

### Zebrafish

All zebrafish experiments were approved by the Washington State University Institutional Animal Care and Use Committee. Larval fish were reared at 28°C in Petri dishes containing water from the Washington State University Vancouver fish facility (900–1000 µS and 7.0–7.2 pH). Transgenic myo6b:GFP zebrafish were used for direct hair cell counts (Kruger et al., 2016). The ty220d *mariner* mutant line (RRID: ZFIN_ZDB-GENO-140707) was used for studies that tested the necessity of functional mechanotransduction on acoustic stimulation-induced hair cell damage (Nicolson et al., 1998). All other experiments were performed in wild-type (*AB) zebrafish.

### Cavitation device

Four 40-kHz ultrasonic transducers (Beijing Ultrasonics) were epoxy mounted to the bottom of a 11.5-l stainless steel canister with a height of 28 cm and outer diameter of 24 cm (McMaster-Carr #4173T37). Input power to two of the transducers was provided by a 300-W ultrasonic generator (Beijing Ultrasonics) to produce the broadband noise stimulus (the other two transducers provided physical stability but were not activated). An inline rheostat (part #RHS20KE; Ohmite) was used to achieve finer control of power output. Fish were housed in a modified 24-well plate containing a 1-cm-thick layer of encased glycerol on the bottom to dampen cavitation energy.

### Hydrophone and accelerometer recordings

The noise stimulus was calibrated using a mini-hydrophone to measure sound pressure (model 8103, Bruel and Kjaer) and a custom-modified triaxial accelerometer to measure particle acceleration (PCB model VW356A12 with *x*-axis sensitivity = 10.42 mV/ms^−2^, *y*-axis sensitivity = 9.65 mV/ms^−2^, and *z*-axis sensitivity = 10.14 mV/ms^−2^). During calibration, the hydrophone was positioned ∼2.5 cm below the water surface at the position where the middle of the modified 24-well plate sits in the water column. The accelerometer was placed on the top surface of the well plate and held in position with clay. Calibration measurements were made at four voltage outputs (0.5, 0.7, 1.0, 1.7 V) from the ultrasonic generator and measured in terms of sound pressure level (dB re: 1 µPa) and acceleration (dB re: 1 ms^−2^). The tri-axial accelerometer measurements in the *x*-, *y*-, and *z*-axes were reported as a combined magnitude vector and calculated as 20 log (√(x^2^+y^2^+z^2^)) similar to Wysocki et al. (2009) and Vasconcelos et al. (2011).

### Acoustic trauma

**Figure 1.**
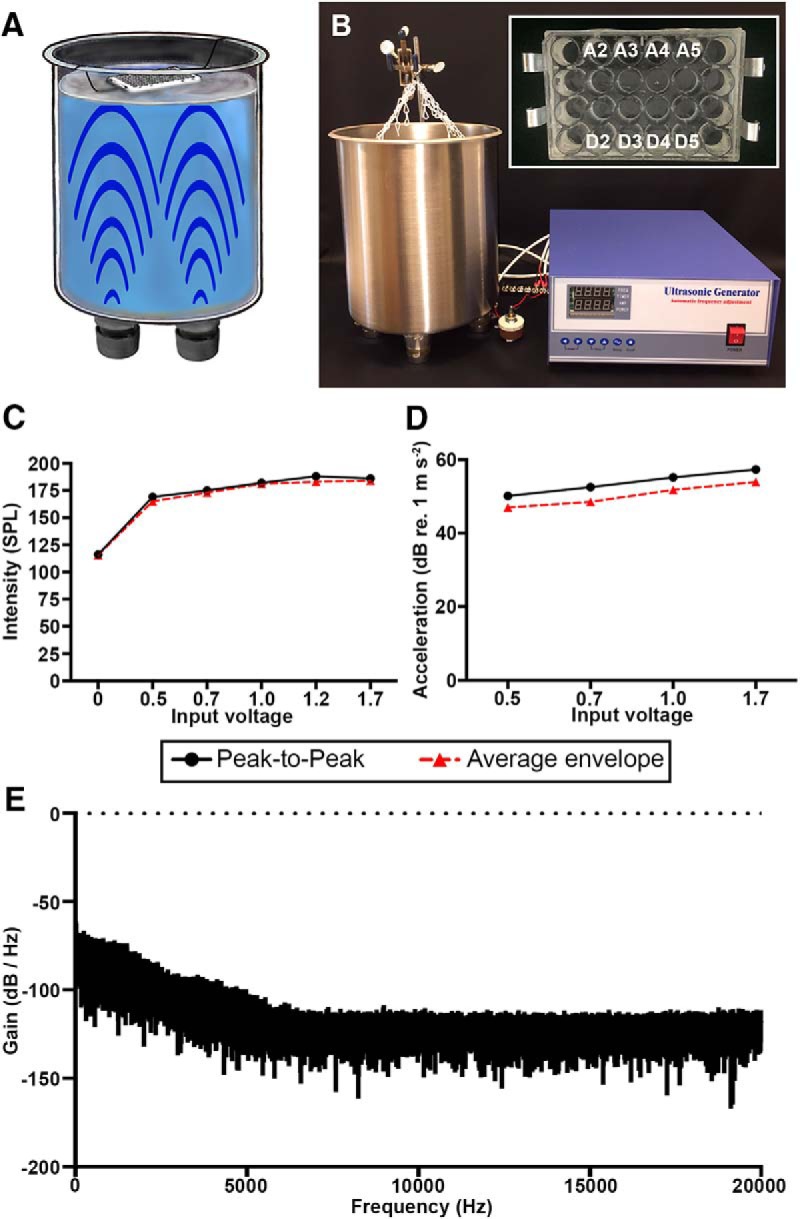
Cavitation device produces intense underwater sound pressure and well plate acceleration. ***A***, ***B***, Two ultrasonic transducers emit stimuli at 40 kHz creating underwater cavitation within the attached stainless steel tank. The signal was generated with a 300-W ultrasonic generator with inline rheostat for fine amplitude adjustment. ***B***, inset, Larval zebrafish are exposed to acoustic stimulation, likely produced by cavitation, in labeled wells of a modified 24-well plate encased in glycerol resting atop 22 cm of water. ***C***, Sound pressure levels measured via hydrophone show increasing intensity with increasing input voltage. ***D***, Peak amplitude of well plate acceleration (measured from the top of the well plate) increases somewhat linearly with input voltage while average envelope exhibits a more modest increase. ***E***, Fast Fourier transform of power spectrum produced by cavitation device shows broadband energy within the low-frequency range.

At 5 d post-fertilization (dpf), fish in a Petri dish were fed with a light dusting of GEMMA micro 75 (Skretting). All noise exposure experiments were conducted at 6 dpf due to the small size and stereotyped neuromast location in this age of fish, which allows for high throughput hair cell assessment. Larval zebrafish were placed into wells A2-A5 and D2-D5 of a modified 24-well plate (three fish per well) containing fish water and suspended atop 22 cm of water with the bottom 1 cm of the plate submerged (Fig. 1). Fish were exposed to an acoustic stimulus for periods ranging from 20 to 120 min. Immediately following acoustic trauma, fish were transferred to a six-well plate (six fish per well) and raised on a 14/10 h light/dark cycle for up to 5 d post-exposure. Following exposure, fish were fed and 50% water changes were conducted daily for the duration of the experiment. Each post-exposure day evening (5–6 P.M.) fish were transferred to a clean six-well plate with fresh fish water.

### Hair cell assessment

Survival of lateral line hair cells was assessed by vital dye labeling in live fish and direct hair cell counts in either live or fixed animals. The vital dye 2-(4-(dimethylamino)styryl)-*N*-ethylpyridinium iodide (DASPEI; Life Technologies) is a marker of mitochondrial membrane potential and stains lateral line hair cells (Harris et al., 2003). Fish were incubated in 0.005% DASPEI for 15 min, rinsed twice with fish water, and anesthetized with 0.001% MS-222 (Argent Labs). Using a Leica M165FC fluorescence dissection scope, 10 anterior neuromasts (IO1, IO2, IO3, IO4, M2, MI1, MI2, O2, SO1, and SO2; Raible and Kruse, 2000) per fish were assessed based on fluorescence intensity (Harris et al., 2003; Coffin et al., 2009). An intensity score of 2 signifies bright neuromast fluorescence, an intermediate score of 1 represents dim DASPEI labeling, while a 0 neuromast score equates to no neuromast fluorescence at a given neuromast’s stereotyped position. The scores from 10 neuromasts per fish were summed such that each fish receives a score between 0 (no neuromast fluorescence) and 20 (full complement of hair cells in all 10 neuromasts). DASPEI scoring is tightly correlated with direct hair cell counts (Harris et al., 2003; Coffin et al., 2013). DASPEI scores were only recorded once per fish and fish were euthanized immediately following neuromast assessment.

Direct hair cell counts were obtained from live myo6b:EGFP transgenic larvae (which express hair cell-specific cytoplasmic EGFP) or fixed *AB or *mariner* mutant fish immunohistochemically labeled with anti-parvalbumin to visualize hair cells. To perform direct hair cell counts in non-transgenic animals, fish were euthanized with an overdose of buffered MS-222 and fixed with 4% paraformaldehyde (PFA) overnight at 4°C. Fish were then rinsed twice with PBS for 10 min each and then once with dH_2_O for 20 min. Larvae were then transferred to blocking solution consisting of 5% goat serum in PBST (0.1% Triton X-100; Sigma-Aldrich) for 1 h. After blocking, fish were incubated in mouse anti-parvalbumin (1:500; EMD Millipore) diluted in 0.1% PBST with 1% goat serum overnight at 4°C (Coffin et al., 2013). Fish were then rinsed three times in 0.1% PBST and incubated for 4 h in Alexa Fluor 488 secondary antibody (Life Technologies) diluted in 0.1% PBST at room temperature (RT). Unbound secondary antibody was rinsed off by three 10-min 0.1% PBST rinses. Labeled fish were stored in 1:1 PBS:glycerol for up to one week before imaging. Hair cells from five neuromasts (IO1, IO2, IO3, M2, OP1) per fish were counted using a Leica DMRB fluorescent microscope.

### Pharmacology

All inhibitors were added to six-well plates immediately after exposed fish were removed from the device. Inhibitors were refreshed during the same intervals as fish water (twice daily) until the end of the desired exposure window. To test the role of protein synthesis we pulse treated fish immediately after acoustic trauma for 4 h with the protein synthesis inhibitor cycloheximide (C7698; Sigma Aldrich). In separate experiments we continuously treated acoustic trauma-exposed fish with either the pan caspase inhibitor Z-VAD-FMK (C7698; Sigma Aldrich) or the antioxidant D-methionine (F7111; UBPBio) to assess the contribution of caspase activation and ROS overproduction, respectively, in the acoustically stimulated lateral line. We also conducted a small blinded screen of select compounds from a larger redox library (BML-2835-0100; Enzo Life Sciences). Compounds chosen had either known protective effects in mammalian models of NIHL (as proof-of-concept) or had not been previously tested against NIHL (to identify new protective molecules; Ohinata et al., 2000; Pourbakht and Yamasoba, 2003; Sergi et al., 2006; Bielefeld et al., 2007; Minami et al., 2007).

### FM 1-43 uptake

We quantified FM 1-43 uptake following acoustic stimulation as a proxy for hair cell mechanosensory activity (Kindt et al., 2012). Briefly, we immersed larval zebrafish in 3 µM FM 1-43FX (F35355; Thermo Fisher) for 30 s followed by four 30-s washes in fish water. Fish were then immediately anesthetized with 0.001% MS-222 and imaged using confocal microscopy with a 40× water immersion objective. Z-stack images of M2 neuromasts were collected and compressed using Leica LAS AF software (Raible and Kruse, 2000). Mean neuromast fluorescence and background fluorescence were measured using ImageJ v. 1.48 (National Institutes of Health). For each image, background fluorescence was subtracted from neuromast fluorescence to yield the reported neuromast fluorescence value (Kruger et al., 2016). Following live imaging of FM 1-43-labeled fish, larvae were fixed in 4% PFA and labeled with anti-parvalbumin for hair cell quantification, as described above.

### Cell death assay

We used a TUNEL assay to label dying cells. Acoustically stimulated fish were euthanized and fixed with 4% PFA (2 h at RT) at various post-exposure time points (0, 24, 48, or 72 h). Fish were then incubated in proteinase K (20 µg/ml) for 10 min followed by a 5 min PBS wash and 5 min in ice cold acetic acid:ethanol (1:2) at -20°C. After two 5-min PBS washes, fish were exposed to 75-µl equilibrium buffer for 30 s followed by incubation in 55-µl working strength TdT for 1 h at 37°C (EMD Millipore S7165). After 1 h, 1 ml of stop wash buffer was added followed by three 1 min PBS washes and 30 min in nnti-dig/block solution. Fish were then rinsed four more times with PBS and immunohistochemically processed with anti-parvalbumin as described above. Three neuromasts (IO1, IO2, and IO3) per fish were imaged on a Leica SP8 confocal microscope. We quantified the number of TUNEL+/parv+ cells per neuromast to determine the number of dying hair cells. We also quantified the number of TUNEL+/parv- cells within a 50 × 50 µm field of view surrounding the hair cells as a measure of cell death in non-sensory cells.

### Synaptic protein labeling

We labeled pre- and postsynaptic proteins in acoustically exposed fish to assess synaptopathy following noise exposure. To label hair cell nuclei, live fish were treated with DAPI (1:1000 in system fish water) for 30 min. Following DAPI treatment, fish were immediately fixed in 4% PFA supplemented with 4% sucrose and 0.2 mM CaCl_2_ at 4°C for 4 h. Fish were then rinsed three times in PBS for 10 min each followed by a 5-min dH_2_0 wash. They were then permeabilized with ice cold acetone for 10 min followed by a 5-min dH_2_0 rinse. Fish were then transferred to blocking solution (0.1% Tween PBST, 1% DMSO, 1% BSA and, 2% goat serum) for 1 h at RT. After blocking, fish were incubated with primary antibodies against the postsynaptic density protein MAGUK (NeuroMab clone K28/86; 1:500) and the presynaptic ribbon synapse protein ribeye b (gift of T. Nicolson; 1:500) overnight at 4°C. Excess primary antibody was rinsed off with four 30-min PBS rinses. Finally, fish were incubated in Alexa Fluor antibodies (1:500) overnight at 4°C. Fish were imaged on a Leica SP8 confocal microscope. To count ribbons, we quantified the total number of ribeye b+ punctae. As a measure of “orphaned” ribbons not associated with a functional synapse, we also quantified the number of ribeye b+ punctae that were not apposed with the postsynaptic marker MAGUK (Sheets et al., 2011).

### Experimental design and statistical analysis

All experiments used animals 6–11 dpf of either sex (sex cannot be determined at this age). Experiments used 8–16 fish per treatment group; animal numbers for each experiment are indicated in the figure legend associated with that experiment. Some experiments were performed blind to control for experimenter bias. Data were analyzed using GraphPad Prism (V. 6.0). Statistical analyses were performed using either an un-paired *t* test assuming equal variance, one-, or two-way ANOVA, as appropriate, and are indicated on each figure legend. *Post hoc* comparisons were performed using Bonferroni corrections. Additional detail is provided in figure legends.

## Results

### Acoustic output of the cavitation device

To stimulate the lateral line of larval zebrafish we developed a novel cavitation device with finely incremented power control. We attached four 40-kHz ultrasonic transducers to the bottom of a water-filled stainless steel tank (Fig. 1*A*,*B*). The transducers were powered by a 300-W ultrasonic generator with inline rheostat that allowed for fine control of power output. During cavitation, zebrafish were housed in a custom 24-well plate encased in glycerol (Fig. 1*B*, inset). Using a hydrophone, we measured sound pressure just beneath the surface of the water bath. We found that both peak amplitude and average sound pressure envelope increased with input voltage up to saturation at 169- and 165-dB SPL (re: 1 µPa), respectively, with a driving power of 1.2 V (Fig. 1*C*). Peak amplitude of acceleration, measured on top of the well plate, increased in a steep linear fashion with voltage input while the increase in average envelope was more modest (Fig. 1*D*). Fast Fourier transform of the power spectrum shows that the stimulus is broadband, with the bulk of the energy concentrated in the low frequency range (Fig. 1*E*). These results demonstrate that our cavitation device produces intense broadband underwater sound pressure and well plate acceleration that can be controlled by adjusting input voltage.

### Acoustic stimulation damages lateral line hair cells

**Figure 2.**
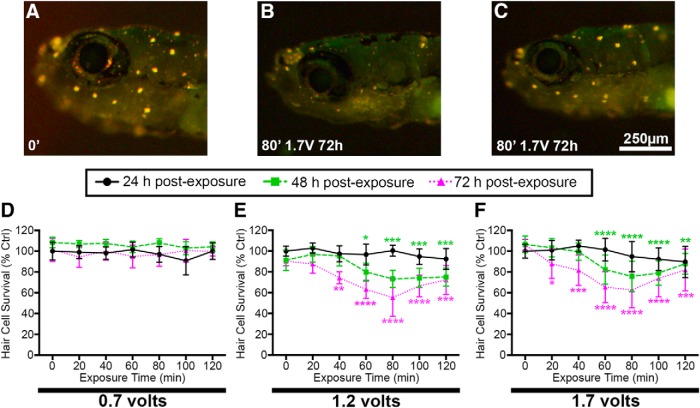
Acoustic stimulation results in exposure time-, intensity-, and post-exposure time-dependent reduction in DASPEI labeling, indicative of hair cell damage. ***A–C***, Representative images of (***A***) unexposed larval zebrafish and (***B***, ***C***) fish exposed to acoustic stimulation. Scale bar applies to all three images. Unexposed fish exhibit bright DASPEI staining indicative of a full complement of lateral line hair cells while fish exposed to 80 min of acoustic stimulation have diminished DASPEI labeling 72 h post-exposure. Two representative images of acoustically exposed fish are shown to depict the diversity of DASPEI labeling observed. ***D–F***, Quantification of acoustic stimulation-induced hair cell loss. ***D***, Fish exposed to 0.7 V show no reduction in DASPEI labeling. ***E***, Fish exposed to 1.2 V of acoustic stimulation exhibit the greatest reduction in DASPEI labeling after 80 min of exposure and 72 h post-exposure. ***F***, 1.7 V of acoustic stimulation produces similar DASPEI reduction to 1.2 V. Asterisks indicate significant differences from age-matched unexposed controls (*p < 0.05, **p < 0.01, ***p < 0.005, ****p < 0.001). Statistical analysis is shown in Table 1. *N* = 10–12 animals per treatment, values are mean ± SD.

To assess the effects of acoustic stimulation on lateral line hair cell survival, we exposed zebrafish to acoustic stimulation and assessed hair cell survival via DASPEI staining. Unexposed controls labeled with the vital dye DASPEI display bright neuromast fluorescence indicative of intact hair cells (Fig. 2*A*). Fish exposed to acoustic stimulation at 1.7 V for 80 min exhibit dimmer DASPEI fluorescence than unexposed controls, indicating that acoustic stimulation reduces hair cell number (Fig. 2*B*,*C*). Acoustically-exposed fish demonstrate variability in hair cell damage, as shown in Figure 2. For example, fish in Figure 2*B*,*C* were exposed to the same damage paradigm but exhibit different degrees of hair cell survival. Fish housed in the device for 8 h with no voltage input had DASPEI labeling consistent with unexposed controls at all post-exposure time points, indicating that handling and housing in the device does not contribute to the decreased DASPEI labeling seen in the acoustically-exposed larvae (data not shown). For the lowest voltage tested (0.7 V), acoustical stimulation up to 120 min resulted in no reduction of hair cell survival for any time points tested (Fig. 2*D*; Table 1). By contrast, 80 min of 1.2-V acoustic stimulation produced a 24% and 39% decrease in hair cell survival for post-exposure times of 48 and 72 h, respectively (Fig. 2*E*). Hair cell survival was lowest for 80 min of exposure and 72 h of post-exposure recovery time. Moreover, the acoustic stimulation induced at 1.7 V produced a similar degree of hair cell damage as that at 1.2 V (Fig. 2*F*). For both 1.2- and 1.7-V driving voltages there was a gradual decline in hair cell survival with increasing exposure time up to 80 min. Interestingly, hair cell survival scores were consistently higher at 100- and 120-min exposure times. This increased hair cell survival may be a result of earlier onset of hair cell death and subsequent regeneration.

**Table 1.** Statistical analysis of hair cell survival, as determined by DASPEI assessment (Fig. 2**) or hair cell counts (Fig. 3)**

Assessment method	Voltage	Variable	*F* score	*p* value
DASPEI	0.7 V	Exposure time	*F*_(12,211)_ = 1.54	*p* = 0.2396
		Recovery time	*F*_(6,211)_ = 1.34	*p* < 0.0001
		Interaction	*F*_(12,211)_ = 1.54	*p* = 0.1131
DASPEI	1.2 V	Exposure time	*F*_(6,216)_ = 25.26	*p* < 0.0001
		Recovery time	*F*_(2,216)_ = 144.0	*p* < 0.0001
		Interaction	*F*_(12,216)_ = 6.23	*p* < 0.0001
DASPEI	1.7 V	Exposure time	*F*_(6,216)_ = 19.72	*p* < 0.0001
		Recovery time	*F*_(2,216)_ = 38.83	*p* < 0.0001
		Interaction	*F*_(12,216)_ = 4.33	*p* < 0.0001
Hair cell counts	1.2 V aLL	Exposure time	*F*_(2,91)_ = 13.39	*p* < 0.0001
		Recovery time	*F*_(2,91)_ = 12.30	*p* < 0.0001
		Interaction	*F*_(4,91)_ = 4.42	*p* = 0.0026
Hair cell counts	1.7 V aLL	Exposure time	*F*_(2,93)_ = 10.87	*p* < 0.0001
		Recovery time	*F*_(2,93)_ = 4.97	*p* = 0.0089
		Interaction	*F*_(4,93)_ = 3.02	*p* = 0.0217
Hair cell counts	1.2 V pLL	Exposure time	*F*_(2,89)_ = 4.90	*p* = 0.0095
		Recovery time	*F*_(2,89)_ = 12.30	*p* < 0.0001
		Interaction	*F*_(4,89)_ = 3.27	*p* = 0.0151
Hair cell counts	1.7 V pLL	Exposure time	*F*_(2,91)_ = 9.85	*p* = 0.0001
		Recovery time	*F*_(2,91)_ = 3.62	*p* = 0.0308
		Interaction	*F*_(4,91)_ = 6.50	*p* = 0.0001

All data were analyzed by two-way ANOVA.

**Figure 3.**
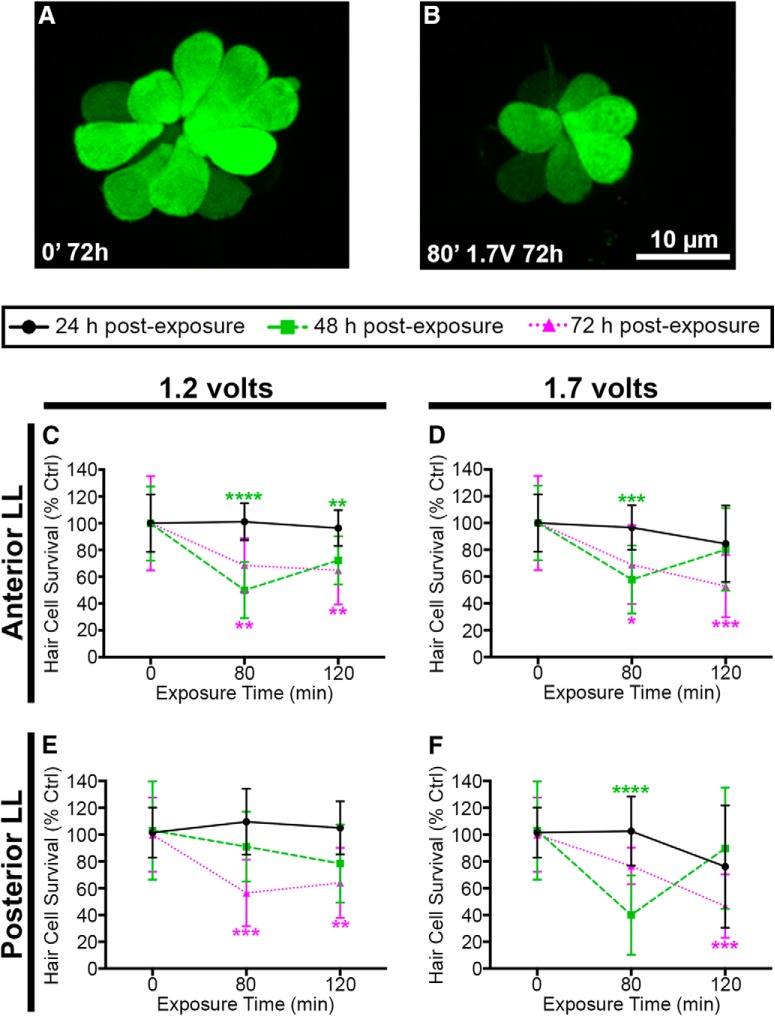
Acoustic stimulation decreases lateral line hair cell number. Unexposed (***A***) and acoustically stimulated (***B***) O2 neuromasts from myo6b:EGFP transgenic larval zebrafish. Scale bar applies to both images. 1.2 V (***C***) and 1.7 V (***D***) of acoustic stimulation significantly reduces the number of hair cells in five anterior lateral line neuromasts at 48 and 72 h after cessation of noise. 1.2 V (***E***) and 1.7 V (***F***) reduces hair cell number in pLL neuromasts P1 and P2. Asterisks indicate significant difference from age-matched unexposed controls (*p < 0.05, **p < 0.01, ***p < 0.005, ****p < 0.0001). Statistical analysis is shown in Table 1. *N* = 10–12 animals per treatment, values are mean ± SD.

To validate DASPEI scores, we exposed transgenic myo6b:GFP fish to acoustic stimulation and obtained direct hair cell counts of GFP-positive cells. Acoustic stimulation caused a significant loss of lateral line hair cells, consistent with the reduction in DASPEI fluorescence (Fig. 3). The acoustic stimulation induced at 1.2 V reduced anterior lateral line hair cell survival at both 80- and 120-min exposure times after either 48 or 72 h post-exposure (Fig. 3*C*). For acoustic stimulation induced at 1.2 V for 80 min, hair cell number was reduced by 50% and 28% for post-exposure times of 48 and 72 h respectively. Exposure to 1.7-V acoustic stimulation similarly reduced anterior lateral line hair cell survival (Fig. 3*D*). Acoustic stimulation at 1.7 V for 80 min reduced hair cell number by 42% and 31% for post-exposure times of 48 and 72 h, respectively. We then examined the effect of acoustic stimulation on two posterior lateral line (pLL) neuromasts (p1 and p2) to determine whether the acoustic trauma effect is consistent across the lateral line system. Both 1.2- and 1.7-V driving voltages caused significant damage in the pLL at 72 h post-exposure (Fig. 3*E*,*F*). Collectively, DASPEI and direct hair cell counts indicate that 1.2- or 1.7-V acoustic stimulation results in robust hair cell damage when assessed 48 or 72 h post-exposure.

### Acoustic stimulation damages saccular hair cells

**Figure 4.**
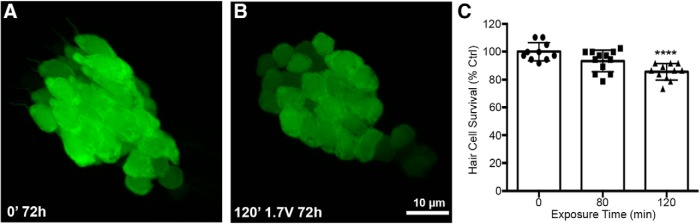
Acoustic stimulation produces an exposure time-dependent reduction in saccular hair cells. Unexposed (***A***) and acoustically stimulated (***B***) saccules from myo6b:EGFP transgenic zebrafish. There was no obvious spatial pattern to the damage in the acoustically exposed saccules. ***C***, Treatment with 1.7 V of acoustic stimulation for 120 min significantly reduces saccular hair cell number when assessed 72 h post-exposure (one-way ANOVA; exposure time: *F*_(2,30)_ = 11.89, *p* = 0.0002). Asterisks indicate significant difference from unexposed age-matched control (****p < 0.001). *N* = 10–12 animals per treatment, values represent mean ± SD.

Adult zebrafish exposed to acoustic trauma exhibit hair cell damage within the saccule, the primary auditory end organ in most fishes (Popper and Fay 1973; Schuck and Smith, 2009). However, Weberian ossicles, which couple the swim bladder to the ear and significantly broaden the detectable frequency range, do not mature until ∼50 dpf, well past the age of the fish used in the present study (Grande and Young, 2004). To determine whether acoustic stimulation damages hair cells in the saccule of larval fish, we exposed myo6b:GFP fish to 1.7-V acoustic stimulation and assessed saccular hair cell number 72 h after exposure. Acoustic stimulation at 1.7 V for 120 min significantly reduced saccular hair cell density by 14% (Fig. 4). This result demonstrates that acoustic stimulation damages saccular hair cells in the larval zebrafish even in the absence of Weberian ossicles.

### Acoustic stimulation does not alter FM 1-43 loading

**Figure 5.**
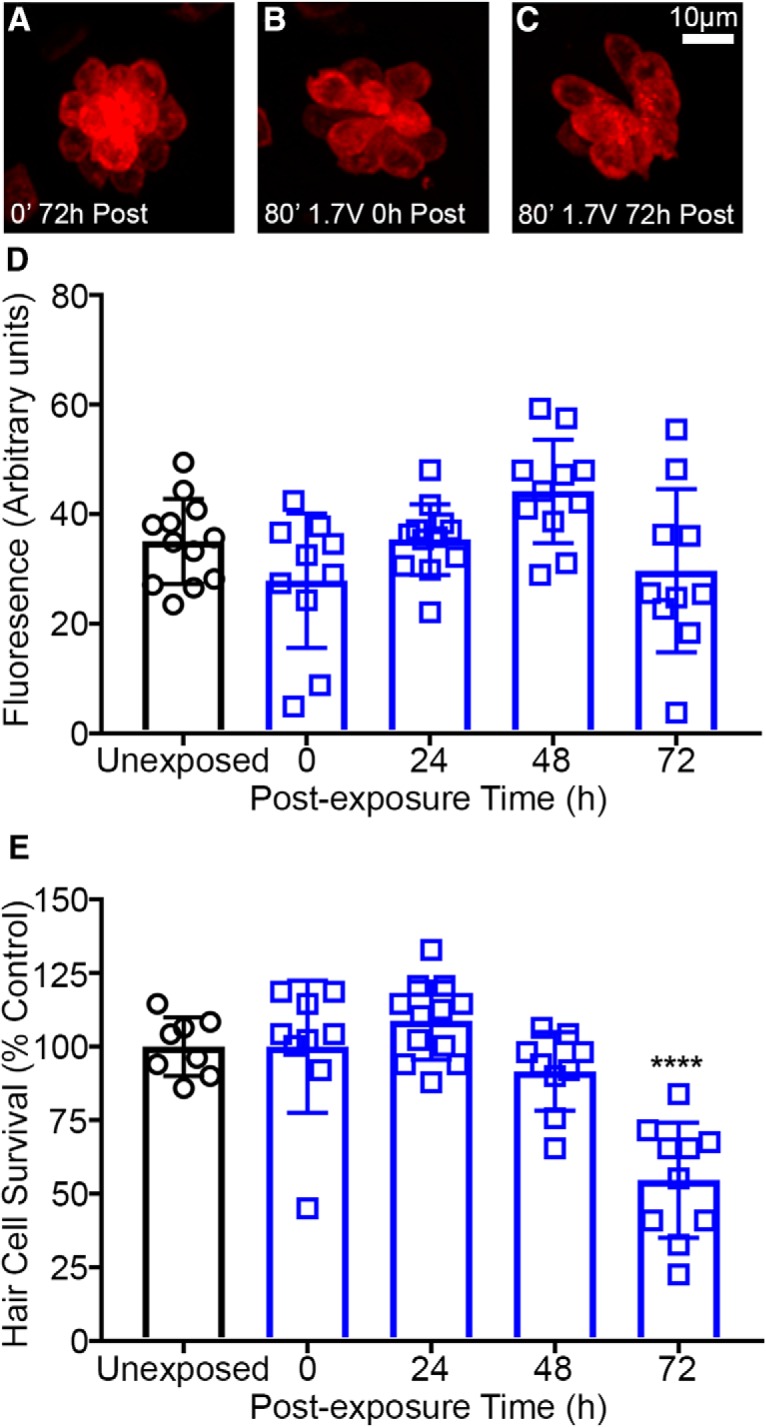
Loading of the mechanotransduction dependent dye FM 1-43FX is not affected by acoustic stimulation in wild-type *AB zebrafish. ***A–C***, Representative images of neuromasts loaded with FM 1-43FX. Unexposed (***A***) and acoustically stimulated (***B***, ***C***) neuromasts are brightly labeled with FM 1-43FX. ***D***, Quantified FM 1-43FX fluorescence (normalized to hair cell number) is not significantly different in unexposed control versus 72 h post-exposure suggesting that acoustic stimulation does not alter hair cell mechanotransduction (one-way ANOVA; post-exposure time: *F*_(4,50)_ = 4.001, *p* = 0.0068). Acoustically-exposed fish exhibit highly variable FM 1-43FX loading. ***E***, Hair cell survival is reduced 72 h after acoustic stimulation (one-way ANOVA; post-exposure time: *F*_(4,43)_ = 17.19, *p* < 0.0001). Hair cells were labeled with anti-parvalbumin and quantified in fixed animals. Asterisks indicate significant difference from unexposed control (****p < 0.001). *N* = 8–12 animals per treatment and values represent mean ± SD.

In birds and mammals, hair cell tip links break from prolonged (4–48 h) exposure to intense noise and rapidly regenerate within 48 h (Pickles et al., 1987; Husbands et al., 1999). To test if acoustic stimulation results in damage to the mechanotransduction machinery, we measured loading of the mechanotransduction-dependent dye FM 1-43 at different post-exposure time points. FM 1-43 loading in acoustically-exposed hair cells was not significantly different from unexposed age-matched controls (Fig. 5). Immunohistochemical labeling of the same fish with parvalbumin demonstrates that this acoustic stimulation intensity was sufficient to cause hair cell loss at 72 h post-exposure (Fig. 5*E*). Hair cell labeling differences may contribute to the delayed onset of hair cell loss when compared to myo6b:GFP (Fig. 3). This result suggests that acoustic stimulation does not damage the mechanotransduction machinery, including tip links, for exposure times up to 80 min.

### Acoustic stimulation decreases hair cell synapse number

**Figure 6.**
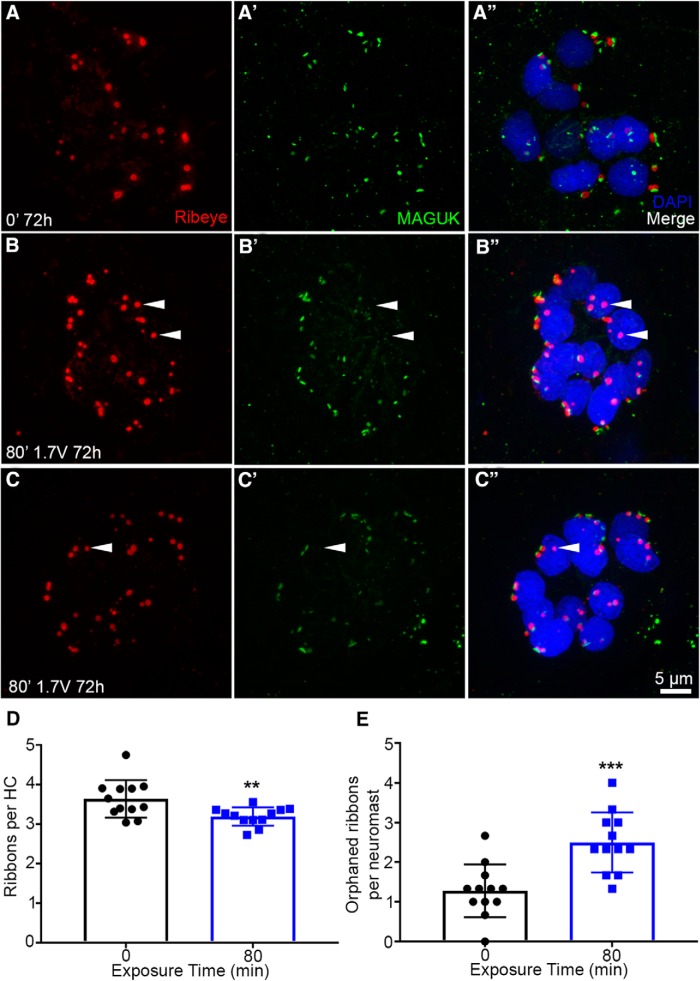
A total of 80 min of acoustic stimulation reduces ribeye puncta and increases the frequency of orphaned ribeye puncta. ***A–A’’***, In unexposed neuromasts, the presynaptic marker ribeye b (red) colocalizes with the postsynaptic marker MAGUK (green). ***B–C”***, 72 h after acoustic stimulation, many orphaned ribeye b puncta are present (white arrows). Hair cells are labeled with DAPI (blue). ***D***, Acoustic stimulation significantly reduces the number of synaptic ribbons per hair cell when assessed 72 h after acoustic stimulation (*t* test; *p* = 0.0079). ***E***, Acoustic stimulation increased the number of synaptic ribbons lacking a neighboring MAGUK puncta (orphaned ribbons; *t* test; *p* = 0.0004). Asterisks indicate significant different from unexposed control (**p < 0.01, ***p < 0.005). *N* = 12 animals per treatment (three neuromasts per animal), values represent mean ± SD.

Cochlear hair cells exposed to moderate noise trauma exhibit a reduction in synaptic ribbons and an increase in orphaned ribbons that are unpaired with a post synaptic density (Kujawa and Liberman, 2009; Sergeyenko et al., 2013). To test if acoustic stimulation leads to synaptopathy in the lateral line we labeled fish with DAPI, presynaptic (ribeye b), and postsynaptic (MAGUK) markers. Colocalization of ribeye b and MAGUK are indicative of synaptic contact. More orphaned ribeye b puncta (white arrows) are present in acoustically stimulated neuromasts than in unexposed controls (Fig. 6). In addition to reduced synaptic coupling, acoustic stimulation also caused a decrease in the number of presynaptic ribbons. Acoustic stimulation for 80 min decreased ribeye b puncta per hair cell when compared to unexposed controls at 72 h post-exposure (Fig. 6*D*). Thus, acoustic stimulation causes a synaptopathic-like phenotype in the larval zebrafish lateral line.

### Onset of acoustic stimulation-induced hair cell death

**Figure 7.**
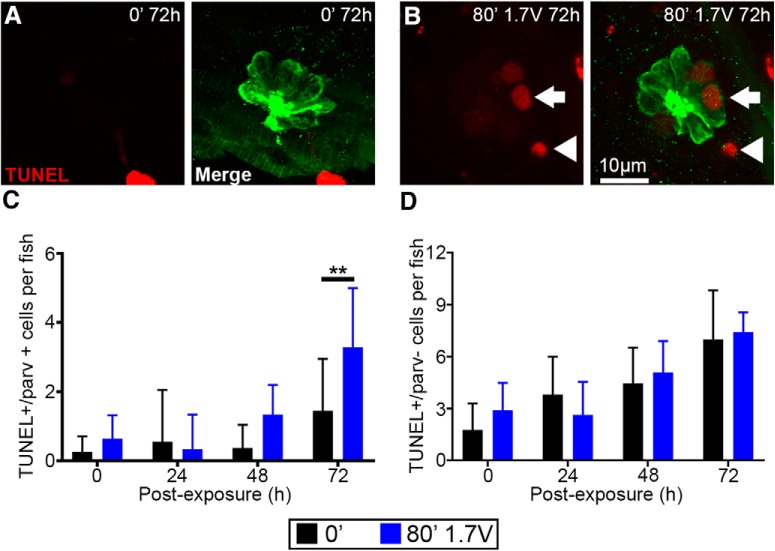
The number of TUNEL+ hair cells increases 72 h after exposure to acoustic stimulation. ***A***, Representative images from unexposed control fish labeled with anti-parvalbumin and processed with the apoptotic maker TUNEL show no TUNEL+ cells within the neuromast. ***B***, 72 h after noise exposure, TUNEL+ hair cells are present within the IO1 neuromast (arrow). ***C***, TUNEL+ hair cells are significantly increased over unexposed controls 72 h post-exposure recovery in IO1, IO2, and IO3 neuromasts (two-way ANOVA; post-exposure time: *F*_(3,71)_ = 12.09, *p* < 0.0001; acoustic stimulation: *F*_(1,71)_ = 9.081, *p* = 0.0036; interaction: *F*_(3,71)_ = 2.872, *p* = 0.0423). ***D***, TUNEL+ parv- cells (non-hair cells, arrowhead) in unexposed and acoustic stimulation exposed fish are not significantly different over 72 h of recovery, suggesting that acoustic stimulation specifically damages hair cells (two-way ANOVA; post-exposure time: *F*_(3,72)_ = 21.91, *p* < 0.0001; acoustic stimulation: *F*_(1,72)_ = 0.3405, *p* = 0.5613; interaction: *F*_(3,72)_ = 1.345, *p* = 0.2666). Asterisks indicate significant difference from unexposed control (**p < 0.01). *N* = 7–12 fish per treatment (three neuromasts per fish) and values represent mean ± SD.

We observed maximum hair cell damage at 48-h post-acoustic stimulation (Figs. 2, 3). To determine the onset and specificity of acoustic trauma we examined TUNEL- and parvalbumin-labeled fish at four post-exposure time points. Unexposed neuromasts had few TUNEL+ hair cells, whereas 1.7 V of acoustic stimulation for 80 min resulted in a 128% increase in TUNEL+ hair cells at 72 h post-exposure (Fig. 7). The chosen post-exposure time points may not fully capture all dying cells, which may explain the lack of TUNEL+ hair cells at 48 h post-exposure. The numbers of TUNEL+ non-sensory cells are similar between unexposed and exposed fish at all four post-exposure times points (Fig. 7*D*). These results demonstrate that hair cells are specifically damaged by acoustic stimulation whereas other non-sensory cell types exhibit similar TUNEL labeling to unexposed controls. Interestingly, TUNEL+ non-sensory cells increase with post-exposure time in both exposed and unexposed fish, which may be due to cell death that has been observed during normal development (Williams and Holder, 2000).

### Hair cell regeneration after acoustic trauma

**Figure 8.**
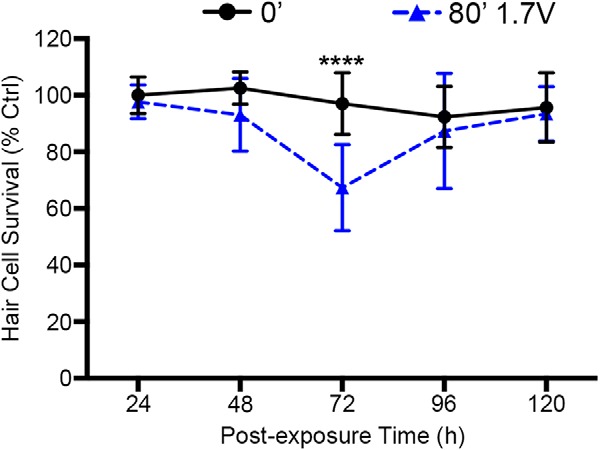
Acoustic stimulation-exposed fish exhibit complete hair cell regeneration. Eighty minutes of 1.7-V acoustic stimulation produces a reduction in DASPEI labeling by 72 h that is completely reversed by 96 h post-exposure (two-way ANOVA; post-exposure time: *F*_(4,99)_ = 7.68, *p* < 0.0001). Asterisks indicate significant difference from age-matched unexposed control (****p < 0.001). *N* = 10–12 animals per treatment, values represent mean ± SD.

Following damage with aminoglycosides, lateral line hair cells completely regenerate within 72 h (Ma et al., 2008). Hair cell regeneration was also observed in the adult zebrafish saccule following acoustic trauma (Schuck and Smith, 2009). We asked if larval lateral line hair cells regenerate following acoustic trauma by assessing hair cell survival with DASPEI every 24 h for up to 120-h post-acoustic stimulation. Following maximum damage at 72 h post-exposure, hair cell survival scores returned to the level of unexposed controls 1 d later (Fig. 8). The extremely rapid regeneration may be accounted for by the staggered onset of hair cell loss, leading to staggered regeneration, unlike the synchronous damage that occurs from ototoxic drug exposure. This observation demonstrates that hair cells regenerate rapidly following acoustic trauma in a timeframe consistent with regeneration from drug insults.

### Mechanotransduction-deficient fish are resistant to acoustic trauma

**Figure 9.**
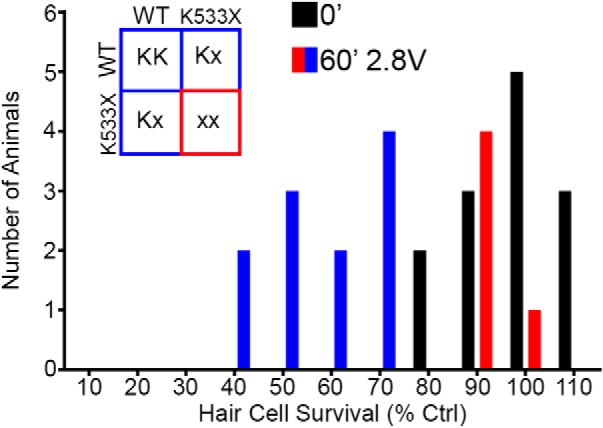
Hair cells in the defective mechanotransduction mutant *mariner* line are resistant to acoustic stimulation damage. Unexposed F1 progeny from *mariner* heterozygotes exhibit DASPEI scores centered on 100%. Exposed F1 fish are distributed in two distinct groups that are similar to the predicted Mendelian distribution (inset), where roughly 25% of fish do not exhibit hair cell damage. *N* = 13–16 animals per treatment, data presented as absolute values.

*Mariner* zebrafish have a defect in myosin VIIa due to a point mutation encoding a premature stop codon, resulting in splayed hair bundles and greatly reduced mechanotransduction (Ernest et al., 2000). *Mariner* mutants exhibit reduced damage to aminoglycoside or cisplatin ototoxicity because these drugs rely on functional mechanotransduction for uptake (Wang and Steyger, 2009; Thomas et al., 2013). We exposed the F1 progeny from heterozygous *mariner* parents to acoustic stimulation to determine whether acoustic damage was reliant on a functioning mechanotransduction apparatus. Hair cell survival scores for exposed F1 progeny were distributed in two distinct groups that match the predicted Mendelian distribution (Fig. 9). Presumed carriers of the homozygous mutant allele exhibited resistance to acoustic damage while siblings carrying at least one copy of the wild-type allele had reduced hair cell survival scores. From this result we hypothesize that acoustic stimulation damage is reliant on a functional mechanotransduction apparatus.

### Caspase or protein synthesis inhibition prevents acoustic stimulation damage

**Figure 10.**
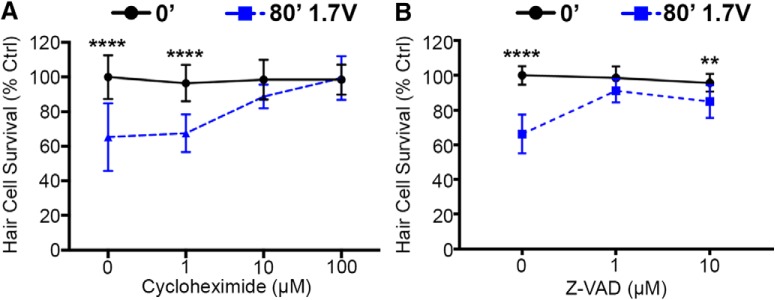
Acoustic stimulation-induced hair cell damage is inhibited by protein synthesis and caspase inhibition. ***A***, A 4-h pulse with the protein synthesis inhibitor cycloheximide immediately after acoustic stimulation reduces hair cell damage when assessed 72 h after acoustic stimulation (two-way ANOVA; cycloheximide: *F*_(3,83)_ = 10.58, *p* < 0.0001). ***B***, 72-h treatment with the pan-caspase inhibitor Z-VAD starting immediately after acoustic stimulation exposure robustly protects hair cells from damage (two-way ANOVA; Z-VAD: *F*_(2,64)_ = 13.9, *p* < 0.0001). Asterisks indicate significant difference from unexposed (0’, 0 µM cycloheximide/Z-VAD) controls (**p < 0.01, ****p < 0.001). *N* = 11–12 animals per treatment, values represent mean ± SD.

The relatively long-time course of acoustic stimulation-induced hair cell damage indicates that protein synthesis may be involved in damage. To test this hypothesis, we pulse treated fish with the protein synthesis inhibitor cycloheximide for 4 h immediately following acoustic trauma and assessed hair cell survival 72 h post-exposure. Cycloheximide treatment protected lateral line hair cells from acoustic trauma in a dose-dependent manner, demonstrating a reliance on protein synthesis for acoustic stimulation-induced hair cell damage (Fig. 10*A*). Acoustic trauma can also result in activation of cell stress pathways that result in caspase activation, leading to hair cell apoptosis (Kurabi et al., 2017). Cochlear hair cells exposed to noise trauma positively label for activated caspases 3, 8, and 9 (Hu et al., 2002; Nicotera et al., 2003). To test the reliance on caspase signaling in our acoustic damage model, we treated acoustic trauma-exposed fish with the caspase inhibitor Z-VAD for the entire 72 h post-exposure window. Z-VAD treatment at 1 µM significantly reduced acoustic stimulation-induced hair cell damage (Fig. 10*B*). These results suggest that acoustic stimulation-induced hair cell damage requires activation of intracellular signaling mechanisms.

### Antioxidants prevent acoustic stimulation damage in the lateral line

**Figure 11.**
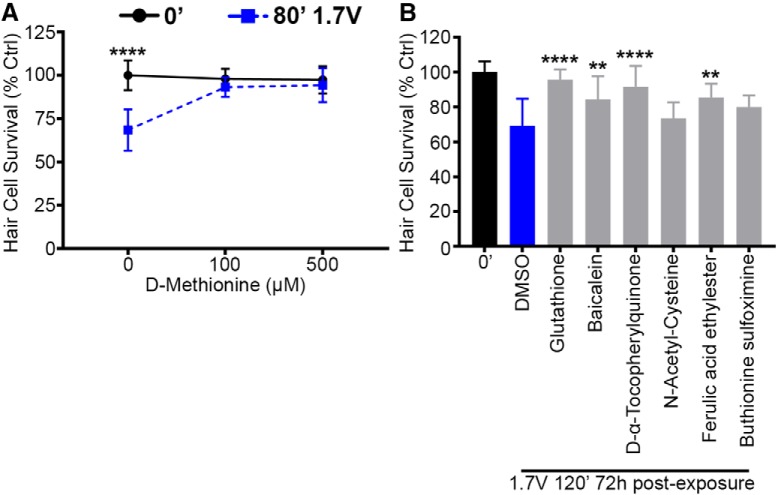
Treatment with antioxidants protects lateral line hair cells from acoustic stimulation. ***A***, 72-h treatment with D-methionine, an antioxidant that prevents NIHL in mammals, robustly protects lateral line hair cells from acoustic stimulation (two-way ANOVA; D-methionine: *F*_(2,69)_ = 14.92, *p* < 0.0001). ***B***, A mini-screen of five antioxidants and the glutathione inhibitor buthionine sulfoximine (negative control) reveals novel hair cell protectants that have hair cell survival scores higher than vehicle (DMSO) control (one-way ANOVA; antioxidant: *F*_(7,82)_ = 12.28, *p* < 0.0001). Asterisks indicate significant difference from unexposed, age-matched controls (***A***) and DMSO-only noise exposed controls (***B***; **p < 0.01, ****p < 0.001). *N* = 11–13 fish per treatment, values represent mean ± SD.

Overwhelming evidence demonstrates that oxidative stress occurs in hair cells exposed to acoustic trauma (Yamane et al., 1995; Henderson et al., 2006; Fetoni et al., 2013). Additionally, antioxidants are the most well studied compound class used to prevent NIHL (Ohlemiller, 2008). To assess if similar mechanisms are present in the acoustically stimulated lateral line, we treated acoustic stimulation-exposed fish with the antioxidant D-methionine, which protects cochlear hair cells from acoustic injury (Cheng et al., 2008; Samson et al., 2008). D-methionine treatment protected lateral line hair cells from acoustic stimulation damage at both 100 µM and 500 µM concentrations (Fig. 11*A*). Finally, we used the zebrafish lateral line as a tool to screen a select redox library for compounds that prevent acoustic stimulation-induced damage. Four compounds (glutathione, baicalein, D- α-tocopherylquinone, and ferulic acid ethylester) significantly protected lateral line hair cells from acoustic stimulation-induced hair cell damage (Fig. 11*B*), only one of which (glutathione) has been previously identified as a potential therapy to prevent NIHL (Ohinata et al., 2000). The pro-oxidant compound buthionine sulfoximine did not confer protection. This result demonstrates the utility of acoustic trauma in the zebrafish lateral line to identify novel compounds that prevent acoustic hair cell damage.

## Discussion

We have designed an acoustic trauma device capable of damaging the larval zebrafish lateral line. This device uses ultrasonic transducers to generate a broadband stimulus within the exposure tank, with the greatest energy in the low-frequency range, where the larval lateral line is most sensitive (Van Trump and McHenry, 2008). Both sound pressure and particle motion (acceleration; Fig. 1*D*) increase with increasing voltage. The device likely causes damage by cavitation; the generation of microbubbles in the fluid medium, which implode, causing localized particle displacement and the generation of pressure waves. As the larval lateral line is a hydrodynamic system, particle displacement is the likely source of the damage, rather than sound pressure (Chagnaud and Coombs, 2014). In the discussion below, however, we describe the stimulus in terms of sound pressure to facilitate comparison to mammalian studies.

We show that acoustic stimulation specifically damages lateral line hair cells in an exposure time-, post-exposure time-, and intensity-dependent manner. Previous work has reported that saccular hair cells from adult fish are damaged by acoustic trauma (Schuck and Smith, 2009), but our study provides the first evidence of acoustic damage in the lateral line and larval saccule using a single exposure method. Our study characterizes the time course of damage and regeneration following acoustic stimulation, demonstrating that maximum hair cell loss occurs 72 h post-exposure, and that significant regeneration occurs by 96 h post-exposure. We also show that acoustic stimulation results in a reduction of ribeye b puncta and an increase in orphaned synaptic ribbons. Using the *mariner* mechanotransduction mutants we show that acoustic trauma relies on an intact mechanotransduction apparatus. Acoustic stimulation results in increased TUNEL+ hair cells, whereas other non-sensory cell types within the neuromast are unaffected, demonstrating that acoustic stimulation damage is specific to hair cells, rather than causing generalized cellular damage. Collectively, these data demonstrate that acoustic stimulation induces damage to the larval lateral line and recapitulates many of the key features of acoustic trauma in the mammalian cochlea.

Acoustic trauma studies in rodents have examined a range of exposure parameters and post-exposure assessment time points that vary based on the degree of desired damage and species (e.g., chinchilla vs mouse). Many mammalian NIHL studies use 1–2 h of continuous noise at 100- to 120-dB SPL (Ou et al., 2000; Hu et al., 2002; Wang et al., 2002). In mice, 100 dB acoustic trauma (1–2 h of exposure time) results in modest hair cell loss [30% inner hair cell (IHC) loss, 50% outer hair cell (OHC) loss], whereas 110 dB (4–8 h of exposure time) resulted in 46% IHC loss and 96% OHC loss (Ou et al., 2000). Chinchillas exposed to 110-dB SPL acoustic trauma for 1 h exhibit as much as 80% OHC loss in the basal region of the cochlea (Hu et al., 2002). In mammals, acoustic trauma results in progressive hair cell loss after cessation of noise, with maximum damage occurring two to four weeks post-exposure (Henderson et al., 1994; Wang et al., 2002). Here, we found that maximum hair cell damage occurred with exposure times of 80–120 min, similar to mammalian NIHL studies. In the lateral line, we observed maximum damage at post-exposure times of 48–72 h, similar to the onset of early stage acoustic trauma in mammals (Wang et al., 2002). However, zebrafish rapidly regenerate damaged hair cells, which may mask assessment of ongoing damage past 72 h post-exposure (Williams and Holder, 2000; Harris et al., 2003).

We also found that lateral line hair cells were damaged by similar sound pressures as mammalian hair cells. Using a hydrophone, we measured sound pressures of 188 and 186 dB (re:1 µPa) for input voltages of 1.2 and 1.7, respectively. It is important to note that our reported values describe sound pressure underwater (dB re:1 µPa). When converted to values in air (dB re:20 µPa), our recorded values correspond to 126.5- and 128.5-dB SPL, similar to acoustic trauma intensities used to induce hair cell damage and permanent threshold shifts in rodent models (Wang et al., 2002; Hirose and Liberman, 2003). These intensities can damage human hearing in as little as 15 min, and we observed a 12% hair cell reduction after just 20 min of the 126.5-dB SPL equivalent (Daniel, 2007). However, it is important to note that mammalian auditory sensitivity is greater than that of zebrafish, particularly larval zebrafish, who lack a Weberian apparatus that functions to increase hearing sensitivity (Higgs et al., 2001). While these differences make it difficult to directly cross compare thresholds for hair cell loss or synaptopathy, we are still confident that our model mimics such components of acoustic trauma and thus allows for the translational study of these phenomena. Collectively, these studies suggest that hair cell sensitivity to acoustic trauma may be conserved across vertebrates.

Adult zebrafish exposed to a 100-Hz pure tone at 179 dB (re:1 µPa) for 36 h exhibit up to 43% hair cell loss in the caudal saccule (Schuck and Smith, 2009). We report less saccular hair cell damage (14%) after just 120 min at 186 dB (re: 1 µPa) broadband exposure. The differences in the degree of hair cell damage are likely due to differences in exposure time, and to the absence of Weberian ossicles in larval fish. Weberian ossicles couple the swim bladder to the ear and first appear around 50 dpf (Grande and Young, 2004). The presence of Weberian ossicles coincides with broadening of the detectable frequency range and lack of this coupling diminishes the fish’s sensitivity to acoustic stimulation (Higgs et al., 2003). The current study finds that, even in the absence of Weberian ossicles, saccular hair cells are damaged by acoustic trauma. The damage observed in larval fish coupled with the ability to image saccular and lateral line hair cells in intact, live preparations makes the larval zebrafish model of acoustic trauma a compelling model for future mechanistic noise exposure studies.

In rodent models, hair cell loss and damage increase as exposure intensity increases (Hu et al., 2000; Wang et al., 2002). We also found intensity-dependent hair cell loss but with an inflection point at 80 min of exposure. We hypothesize that, for exposure times longer than 80 min, the onset of hair cell loss occurs rapidly and initiates regeneration while cell death is still occurring, thus masking the maximal effect. To test this hypothesis, future studies can use fate-mapping strategies in the neuromast to differentiate hair cells that were present before acoustic stimulation from newly regenerated hair cells (Suli et al., 2014; Cruz et al., 2015).

Cleaved caspases are present in cochlear cells after acoustic trauma and intracochlear perfusion of the caspase inhibitor Z-VAD reduces hair cell loss in guinea pigs following blast noise (Hu et al., 2002; Nicotera et al., 2003; Abaamrane et al., 2011). We found that Z-VAD completely prevented acoustic trauma in the lateral line. Similar degrees of protection were reported in some mammalian studies (Abaamrane et al., 2011), while other studies find that Z-VAD treatment provides only partial attenuation of acoustic trauma in rodents (Zheng et al., 2014). While the magnitude of protection likely depends on the precise acoustic stimulus employed and the method of Z-VAD delivery, the collective data suggest that reliance on caspase cleavage is conserved between rodent and zebrafish models of acoustic trauma. Differential gene expression patterns have also been identified in noise-exposed cochlea (Cho et al., 2004; Kirkegaard et al., 2006; Yang et al., 2016). The importance of protein synthesis in acoustic trauma is difficult to test in rodents due to the high systemic toxicity of protein synthesis inhibitors (Hoffmann et al., 2010). Zebrafish offer the advantage of treatment via bath-applied inhibitors, which can easily be pulsed to prevent chronic toxicity. We found that treatment with cycloheximide for 4 h after acoustic stimulation prevented hair cell loss when assessed 72 h later, suggesting that acoustic trauma-induced hair cell damage relies on protein synthesis. These studies demonstrate the ability of the lateral line to elucidate mechanisms of acoustic trauma.

Acoustic trauma results in ROS overproduction in the cochlea that persists for up to 10 d (Yamane et al., 1995; Vlajkovic et al., 2010). Many antioxidants have been reported to reduce NIHL in rodent models (Ohlemiller, 2008). For example, the antioxidant D-methionine protects cochlear hair cells in rodents from acoustic trauma (Cheng et al., 2008; Samson et al., 2008). However, a clinical trial using N-acetylcysteine to prevent NIHL showed some promise but failed to meet clinical endpoints (Kopke et al., 2015). In the present study, we report that D-methionine completely protects lateral line hair cells from the damaging effects of acoustic stimulation. Such robust protection is not regularly observed in rodent studies, likely because those studies often use systemic injection of antioxidants, rather than the direct immersion method employed here (Samson et al., 2008; Clifford et al., 2011). However, antioxidant protection from acoustic trauma is clearly conserved between fish and mammals. Zebrafish are a productive tool to identify new compounds that prevent drug-induced hair cell death, with some compounds demonstrating protection in mammalian models of ototoxicity (Owens et al., 2008; Vlasits et al., 2012; Chowdhury et al., 2018). Building on the success of these otoprotective screens, we performed a phenotypic hair cell protection screen of a redox library and identified three novel protective antioxidants and confirmed the protective potential of glutathione. Future work will expand on this screen to identify additional novel compounds that prevent acoustic trauma-induced hair cell death with the goal of developing preventative therapies.

The discovery of cochlear synaptopathy transformed our understanding of sound levels that were previously thought to be innocuous (Kujawa and Liberman, 2009). The phenomenon of synaptopathy appears to be well conserved across species. Synaptopathy has been observed in rodents and nonhuman primates and its existence in humans is supported by some physiologic evidence (Liberman et al., 2016; Valero et al., 2017). The characteristic phenotype of synaptopathy includes a decrease in ribbon synapse density and an increase in isolated ribbon synapses that lack a paired postsynaptic density (Sergeyenko et al., 2013). Zebrafish studies using direct application of AMPA suggest that this damage is mediated by calcium permeable AMPARs, however, mechanistic studies with physiologically relevant stimuli are needed (Sebe et al., 2017). In the present study we demonstrate that acoustic stimulation likely produced by cavitation results in decreased ribeye b puncta and increased orphaned ribbons, indicative of synaptopathy. Our data are consistent with synaptic changes in noise-damaged cochlear hair cells and allow for future mechanistic dissection of synaptopathy using physiologically relevant stimuli.

While the zebrafish lateral line cannot model all of the features of cochlear injury (e.g., stria swelling), we demonstrate that use of this model allows us to isolate hair cell-specific responses to acoustic trauma using an *in vivo* preparation. In summary, our study demonstrates that our novel cavitation system represents an accessible *in vivo* model for accelerating our understanding of acoustic trauma and developing protective therapies.
